# Developing an AI-Enabled Integrated Care Platform for Frailty

**DOI:** 10.3390/healthcare10030443

**Published:** 2022-02-26

**Authors:** Angelina Kouroubali, Haridimos Kondylakis, Fokion Logothetidis, Dimitrios G. Katehakis

**Affiliations:** Foundation for Research and Technology Hellas, Institute of Computer Science, 70013 Heraklion, Greece; kondylak@ics.forth.gr (H.K.); fokion@ics.forth.gr (F.L.); katehaki@ics.forth.gr (D.G.K.)

**Keywords:** integrated care, digital health, eHealth, frailty, elderly, informal care, artificial intelligence and big data technologies

## Abstract

Informal care is considered to be important for the wellbeing and resilience of the elderly. However, solutions for the effective collaboration of healthcare professionals, patients, and informal caregivers are not yet widely available. The purpose of this paper is to present the development of a digital platform that uses innovative tools and artificial intelligence technologies to support care coordination and shared care planning for elder care, with a particular focus on frailty. The challenges of shared care planning in the coordination of frailty care are demonstrated, followed by presentation of the design and technical architecture of an integrated platform. The platform incorporates all elements essential for the support of daily activities, coordinated care, and timely interventions in case of emergency and need. This paper describes the challenges involved in implementing the platform and concludes by reporting the necessary steps required in order to establish effective smart care for the elderly.

## 1. Introduction

One of the main achievements of modern society is longevity. In 2020, more than one fifth (20.6%) of the EU population was aged 65 or above [1]. In combination with low birth rates, longevity is expected to bring significant changes to the structure of European society. Population ageing has profound implications for the planning and delivery of health and social care. EU spending on medical care is currently growing faster than the gross domestic product (GDP) [1,2]. According to the 2015 Ageing Report, the total ageing costs in the euro area are projected to increase by 1.5 percentage points of GDP, from 26.8% in 2013 to 28.3% in 2060 [3]. Health spending is growing faster than economic growth, mainly due to an aging population, rapid urbanization, and sedentary lifestyles. Between 2000 and 2017, global health spending increased by 3.9% per year, while global GDP grew by 3.0% [4]. As chronic diseases are related to age, the older someone gets, the more likely it is for him or her to develop at least one chronic condition such as hypertension, diabetes, or rheumatoid arthritis. In addition to chronic diseases, frailty is a very common clinical condition for the elderly.

### 1.1. Frailty

Frailty is a dynamic state of heightened vulnerability to stressors and has a multidimensional nature [5]. Frailty leads to a spiral of decline in various functional domains, including the following four general categories: (1) physical frailty (unintentional weight loss, self-reported exhaustion, weakness, slow walking speed, and low physical activity); (2) cognitive frailty (distinct clinical concept of simultaneous physical frailty and cognitive impairment in the absence of concurrent dementia); (3) psychological frailty (loss of resilience in cognitive, mood, and motivational components); and (4) social frailty (loss of social resources and behaviours that are important for an individual’s social needs) [6]. Frailty is usually associated with the risk factor of loneliness. Elderly people who experience high levels of loneliness are at an increased risk of becoming physically frail.

Frailty has no single dependence on chronic diseases. Poor clinical outcomes are related to a depletion of homeostatic reserves and resilience that reduce the capacity to compensate ageing-related molecular and cellular damage. When ageing, loneliness and frailty coexist, multiple health and care interventions are required. High specialization of hospital and home care are required in order to address frailty care, which brings to the surface the constant challenge of improving the efficiency and quality of healthcare provision. Integrated care involves creating a holistic and innovative approach to assessing and addressing varied needs, encompassing the spectrum from physical assessment to mental health and psychosocial risk factors. While it offers the opportunity to preserve function and activity in late aging, health systems are currently organized to identify and treat acute illness, not deliver integrated care [7].

Frailty care has moved away from the medical paradigm that focused on its treatment as a biological syndrome towards the adoption of a broader, multi-dimensional approach in order to acknowledge psychological elements such as quality of life as well as social elements such as lack of social contacts, situational factors, and wellbeing. Preventive strategies include innovative methods for screening and identification of pre-frail status in older adults. To promote preventive strategies and support integrated care for the elderly it is necessary to introduce the process of shared care planning in order to facilitate communication between patients, clinicians, social workers, and informal caregivers [8].

### 1.2. Shared Care Plans

Shared care plans (SCP) support connected and patient-centred long-term health care. A shared care plan is based on a patient-centred health record that can be shared by many members of a care team, including the patient and their caregivers. SCPs help to improve relationships, understanding, and efficiency. They are especially useful for patients with chronic illnesses and conditions, whose health coordination is often particularly complicated. The SCP concept can only be used when health and social care services are organized in a collaborative manner and professionals cooperate in the provision of elder care. However, this is an organizational issue, not a technological one.

SCPs support several important health outcomes, including communication between patients and their health teams, coordination between health providers, increased transparency, patient self-management, patient engagement, goal-setting, provision of timely information, quality of care, and shared decision making [9].

On the other side of the equation, older adults are faced with an overly complex range of choices in terms of screening, detection tests, and/or treatment modalities, which can neglect important issues such as psychosocial complications. Oder adults often experience decisional conflict, anxiety, worry, and frustration. Decisional conflict occurs when individuals experience uncertainty about which course of action to take when choice among competing options involves risk, loss, regret, or challenge to personal life values [10]. Distress and tension are the first obstacles to informed and responsible decisions, and are often due to lack of knowledge. Patient decision aids have been shown to consistently improve knowledge, reduce decisional conflict, and result in choices congruent with patient values, thereby promoting patient empowerment [11,12].

Caring for older people requires a holistic approach. Informal care is a fundamental part of the integrated care continuum and plays an important role in the wellbeing and resilience of the elderly [13]. As the focus of healthcare delivery shifts from hospital care to maintaining an active and healthy life, resources such as time, money, and attention are increasingly being allocated from the end of the healthcare value chain (treatment and aftercare) to the beginning (prognosis, prevention, and early diagnosis). People have already begun to play a more active role in the prevention, treatment, and monitoring of their health. The COVID-19 pandemic has further reinforced the need for care outside of the traditional routes in order to ensure that the health system is decongested and safety is preserved.

### 1.3. Platform Challenges in Elder Care Services

For the delivery of an Elder Care platform capable of supporting integrated health care delivery there are several challenges that should be appropriately addressed; the barriers that hinder active and healthy ageing must be tackled by changing the way the user experiences the services provided through the processes involved. An overall technological solution that is reliable, efficient, holistic, and responsive and that will promote individual health management and active participation in individualized care planning is required. In this direction, the establishment of monitoring and performance evaluation systems to support the ongoing paradigm shift in the provision of elder health and care management towards more appropriate and goal-directed smart services is an important first step. Such goal-driven services can enable the adoption of integrated pathways of care that are more efficient, less costly, and enable the continued autonomy of elderly persons.

Uninterrupted sharing of information is required among all involved stakeholders. Information sharing enables communication across service boundaries while at the same time preserving privacy and facilitating formal and informal care, reducing unnecessary visits to health providers, and improving patients’ sense of security. An appropriate alert system is essential as well in order to prevent and better manage critical events. In cases such as acute episodes, alerts contribute to the acceleration of the emergency handling response while promoting older adults’ sense of safety at home despite any potential limitations they may have due to health conditions [14].

Furthermore, psychological and emotional support is required in order to mitigate psychological and emotional distress, loneliness, and isolation and to avoid social exclusion and support mental health and independent living while promoting a healthy lifestyle and safe habits. End-user education and training can significantly aid this purpose, promoting a sense of security about dealing with individual health status and gradually improving overall psychological wellbeing.

Closely related to education and training is user engagement and technology acceptance as concerns the available information technology (IT) solutions. Older adults may face difficulties in using certain technologies, as they may have a low level of digital proficiency and skills. Enabling smart health through unobtrusive health sensors and the internet of medical things has the potential to enable unobtrusive health monitoring even for elderly persons who are not digitally skilled via appropriate medical practice, training, and education.

Further, patient medical data generally remain in health silos and cannot be readily integrated, homogenized, or exploited. If appropriately combined, homogenized and used, data analytics and artificial intelligence (AI) technologies (through advanced machine learning and big data techniques) can be used to obtain better understanding of disease progression, psychosocial factors, gender dimensions, and the correlations of the same with disease progression in order to formulate a solid knowledge base that will promote disease understanding and move health care provision forward. As such, enhancing the interoperability, flexibility, and scalability of potential solutions is necessary in order to overcome technology silos and embrace architectures mature enough to permit rejection of one-size-fits-all frameworks, allowing for global health data exploitation in an efficient and cost-effective way [15,16].

The remaining portion of this paper is structured as follows: in Section 2, we present the methodology used to create the integrated platform; in Section 3 we present the architecture and modules of the Elder Care platform; in Section 4, we discuss implementation of the platform in the home care setting and the challenges that often accompany technology implementations for the elderly; finally, Section 5 concludes the paper.

## 2. Materials and Methods

The methodology for the design and implementation of the Elder Care platform, presented in this paper is based on ISO/IEC 12207:2008 (Systems and software engineering: Software life cycle processes). The software implementation strategy was based upon an iterative and incremental development life cycle model [17]. This was selected as the most appropriate method to address the complexity of the proposed solution and its implementation. The methodology included a solution design phase, a prototyping phase, and a pilot implementation phase. The solution design phase identified the user requirements in order to provide details concerning the development of the proposed solution. The Elder Care platform described here is designed for usability and adaptability. The iterative design process adopted user-centred (UCD) and user-driven (UDD) design approaches and philosophies in order to analyse and envision the ways in which users are likely to use the solution as well as to validate our assumptions with regard to user behaviour. Outcomes in this stage included a clear and feasible plan as to how to successfully develop both a solution and a clear and feasible business model with an exploitation and commercialization plan. Prototyping was beyond the scope of this paper.

The Elder Care platform presented here is an innovative information and communications technology (ICT) platform that aims to address the varied needs of the elderly people spanning physical conditions, mental health, and psychosocial wellbeing using specific methods for screening and identification of pre-frail status. The platform has several elements, including:

- A mobile app for older adults and informal caregivers and a web app for healthcare professionals.
- Frailty and pre-frailty assessment and detection to prevent disability, avoid adverse health events, and hospital admissions.
- Social and healthcare system integration incorporating understanding of psychosocial factors and the gender dimension as well as correlations which may affect frailty progression.
- Compliance with ethical, legal, and regulatory requirements, including the general data protection regulation (GDPR) [18].
- Interoperability capabilities to connect with third party digital systems. The platform follows the interoperability-by-design principle based on international standardization efforts and best practices. It focuses on supporting the continuum of care to better support elder needs, to build and communicate meaningful information across service boundaries, and to facilitate better management of resources and provision of services. More specifically, the platform can connect to existing standards-compliant health infrastructure (e.g., hospital information systems and electronic health records) to support access to available citizen data.
- Frailty management support for the delivery of more user-centred care interventions with the aim of slowing the impact of frailty on cognition, mental health, autonomy, and quality of life.
- Facilitation of user empowerment towards self-management and autonomy through (amongst other factors) the education and training of the care team, including informal caregivers.
- User experience improvement through a rigorous monitoring and performance evaluation system intended to improve the provision of goal-directed services and facilitate data collection for research purposes in order to connect the results into an evidence-based roadmap.
- User-centred techniques appropriate for the profiles of the older adult users who may be pre-frail and feeling lonely and/or isolated.
- Adaptiveness to user needs and to their level of familiarity with new technologies.
- Services to mitigate feelings of loneliness and isolation, avoid social exclusion, and support mental health and independent living.
- Promotion of healthy lifestyles and safe habits.
- Sustainable and affordable in terms of purchasing price, maintenance, and operating costs.

Holistic frailty screening is necessary to enhance the prevention and management of frailty. Digital tools can provide innovative solutions focused on older adults, facilitating the implementation of a shared care plan with the health and care team. The Elder Care platform presented here is a set of tools centred around the shared care electronic health record (EHR) of an older adult individual. It was created using a modular approach to support the described challenges in an integrated and seamless manner, offering an interface adaptable to the particular needs of each user type [12]. The end user applications were developed for older adults and their care team in order to support multidimensional goal-directed interventions, and were designed to provide feedback to older adult users as well.

## 3. Results

The COVID-19 pandemic crisis has stressed that there is often no viable alternative to remaining at home. However, solutions for the effective collaboration of healthcare professionals, patients, and informal caregivers are not yet widely available, and usually focus on information exchange between patients and health personnel [19]. This results in the duplication of efforts and costs and in overall heterogeneous accessibility of caring [20]. Recent approaches have been fragmented, usually focusing only on a specific disease or on connecting patients with healthcare professionals, and neglecting to some extent the role of informal caregivers [13].

The recent health crisis has pointed out obstacles that need to be overcome in order to truly move the implementation of digital tools towards highly coordinated, physician-guided, and patient-involved medical care [21]. Examples of emerging digital health trends amplified by the ongoing COVID-19 pandemic include the rise of telemedicine, telehealth, remote monitoring of patients, elder care, the development of national digital health portals using big data, cross-border digital health cooperation, applications, wearables and self-monitoring solutions, and tools to facilitate access to patient data by patients.

In this paper we present a design and implementation plan for an integrated care platform that builds on an AI and Big Data-enabled and high technology readiness level state-of-the-art personal health framework (PHR-C) [22] to enable shared care plan processes and informal care coordination [23]. PHR-C has been implemented through various research and development projects focusing (amongst others) on the management of pain and stress [24], COVID-19, and cancer [12]. Platform services offer intelligent communication and effective management of interconnections of patients, professionals, and informal caregivers, exploiting advanced AI algorithms to support routine activities. The Elder Care platform extends a personal health record system to support the coordination of care through a shared care plan-focused design.

The Elder Care platform provides the possibility of informal caregivers to accessing the platform as a stakeholder for the shared care plan. Informal care is often left outside of integrated care services planning. This platform accounts for the involvement of informal caregivers in order to help them take care of elderly persons in a more efficient manner. Access to the platform is provided upon receipt of consent from person receiving care. It builds on an existing personal health system and addresses the specific challenges involved in providing a complete care coordination system.

**Figure 1:**
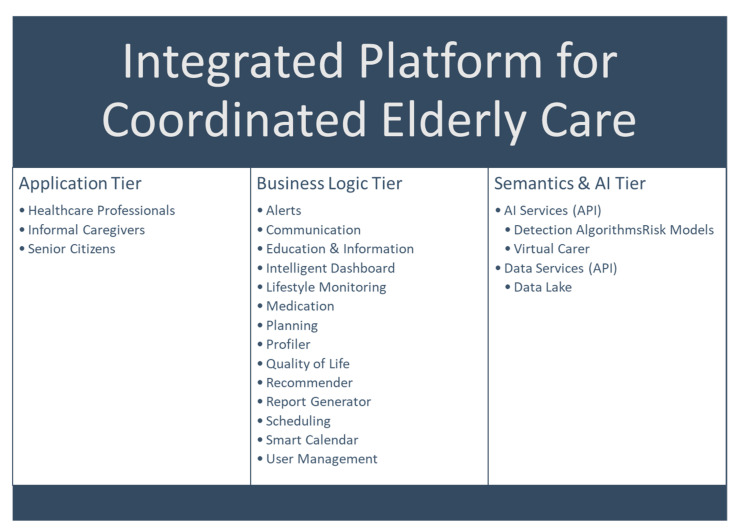
High-level depiction of the core components of the proposed solution. Security, integrity, and interoperability services are horizontal to all tiers. Interoperability Services in particular aim to link data originating from wearables such as smartwatches, EHRs such as those based on national infrastructures, sensors (e.g., for temperature measurement), medical devices, and other databases.

The high-level architecture of the solution is shown in Figure 1, and consists of the Application Tier, the Business Logic Tier, and the Semantics and AI Tier. In this section, we describe each of these layers in detail, as well as the required Interoperability Services which are horizontal to all layers. The platform integrates different modules developed in various research and development projects. Each module is at a different technology readiness level (TRL). The overall platform is at TRL 6, meaning that the technology was demonstrated in a relevant environment (industrially-relevant environment in the case of key enabling technologies); however, the actual deployment in a fully-operational environment is work in progress.

### 3.1. The Application Tier

The Application Tier components are mobile apps for patients and informal caregivers that run on Android and iOS smartphones, as well as a website for healthcare professionals which allows intelligent patient data visualization and user role management. In addition, the services communicating with the mobile and web apps are implemented as individual RESTful services in full compliance with the proposed client platforms. The app for health professionals can be easily accessed through a hospital portal if such a portal is available.

### 3.2. Business Logic Tier

The following models are available at the business logic tier.

**User Management**: The Elder Care platform differentiates user types and can include new users in the system, including older adults, their caregivers, formal caregivers, healthcare professionals, and social workers. An administration panel enables the creation of new healthcare professional and/or social worker profiles. Healthcare professionals and/or social workers can then register new older adults and their caregivers into the platform. The user management module supports access to different application modules based on user type, linking different user accounts for access to clinical data, setting up initial patient configuration by the administrator (i.e., language, demographics, health professional, caregiver, planned surgery), and auditing. In addition, access to information is regulated by the patient her/himself, who is able to easily identify who has access to what and to grant or deny access to her/his data. The older adult, through an administrator or by her or himself, can add many informal caregivers or family members that can participate in data collection. In addition to manual data entry by the older adult and by formal and informal caregivers and family members, the system reuses existing information (e.g., EHR) as much as possible along with local authentication techniques and/or biometric signatures.

**Profiler:** this module gathers relevant information from older adults, including health and psychosocial needs, preferences and limits, knowledge, habits, socialization, attitudes, and capability in order to adapt the stored information to individual needs and preferences [25]. This module collects relevant information on the optimization and development of the shared care plan. The shared care plan is established in collaboration with healthcare and social care providers responsible for the care of each older person. As such, it enables the collection of information that will guide the selection of the services that are needed for each individual in order to best serve their needs, preferences, and limitations. All necessary actions are taken to assure equality of use and to avoid age or gender discriminations. Data from a personal profile are combined with monitoring data and implicit and explicit feedback provided by formal and informal caregivers and family members when using the app to update patient information and tailor the best coaching strategy in accordance with professional health support.

**Quality of life**: this module gathers data and information through questionnaires and unobtrusive monitoring, which is necessary to identify psychosocial, cognitive, physical, and frailty status. The Elder Care platform can integrate third party commercial solution components that gather information about the quality of life of the user on a daily basis, using a virtual caregiver, sensors, medical devices, validated self-reported questionnaires, and constant monitoring of daily life and habits. Data gathered in this module are used to deliver the frailty risk assessment of older adults. Specifically, for the quality-of-life questionnaires the sampling frequency can be preset according to the expert advice of health professionals/social workers. A rule-based system allows healthcare and social care providers to define when each data item should be collected in order to identify potential signs of frailty. The care providers can then propose a treatment plan to help elderly people prevent frailty.

**Virtual Caregiver**: a virtual caregiver intelligently engages with the user based on its prediction of their emotional/psychological status; the collected data can enter a feedback loop in order to update the Virtual Caregiver attitude. The Virtual Caregiver relies on both data collected by itself and on data collected by sensors, other devices, self-reporting, and monitoring of sleep quality, stress, significant life events, physical activity, and quality of social interactions. All collected data are analysed using state-of-the-art machine learning in order to provide older adults with tailored support leveraged by a better understanding of the unique factors and behaviours associated with cognitive decline, loneliness, social isolation, resilience decrease, and behavioural symptoms. These algorithms complement the detection, classification, and prediction of early pathological cognitive decline in older adults, and require the use of novel inputs such as wearables, mobile devices, and sensor signals.

In addition to assessment, the virtual caregiver can be used to improve the psychosocial and physical state of the user. It delivers the news of the day, provides new learning opportunities, and can offer books or audio books, notify the user about events in his/her smart calendar or nearby cultural events, talk about daily tasks, and offer guidance for simple relaxation exercises such as deep breathing, progressive muscle relaxation, guided images, and mindfulness. It offers overnight tools to aid sleep and to beat insomnia as well. Overall, it provides basic emotional support and helps the user to address their immediate basic needs and find information services and psychosocial events. In case of critical events, care professionals are alerted in order to address a fast response, avoiding future complications resulting from emotional or psychological distress from that same critical event. This support is provided either in person or by phone/video-call triggered by the virtual caregiver.

**Communication**: this module supports synchronous and asynchronous communication between older adults and their care team, as well as between older adults for social purposes, by exploiting a messaging system. It provides a dedicated channel to exchange information and communicate regularly, including preset messages based on specific older adult needs. This module allows users to mark other community members as friends, enabling asynchronous communication.

In addition, the Elder Care platform includes a **user helpdesk**. Older adults with concrete questions are able to use a virtual agent to issue a question, while care providers can either use the web or their mobile device to exchange messages and contact the professional care team in case of emergency. Predefined messages can be configured to be sent to users at specific time points based on the shared care plan.

**Planning:** this module enables the setting up and tracking of such goals and targets for physical activity, nutrition, blood glucose and other vital sign levels, weight, and emotional regulation and socialization targets (e.g., presence at events). Exploiting the information collected by the profiler, existing care pathways are matched with user profiles by identifying the services that can be delivered in a holistic, anticipative, and based-on-function type of care in order to address users’ needs, preferences, and possible limitations. Then, this module supports the coordinated action of team members toward a common goal to provide quality integrated care. An overview of the patient information and corresponding plan is conveyed in the shared care plan dashboard (intelligent dashboard and analytics module). Based on the type of user, different views of the planning module are presented to the older adult, the caregiver and the care professionals. The care professionals are allowed to change the care plan, whereas older adults and their caregivers can enter requests for change. Adjustments in the targets are performed by the care professionals, ensuring that they reflect the older adult’s current health status. Upon change, an automatic notification is sent. Both older adults and care professionals can view progress. Motivation is enhanced through positive messages.

**Recommender:** this module sends personalized pieces of advice about how to reach the level of health behaviours that each user requires. This module is directly linked with the lifestyle monitoring and planning modules. The recommender uses machine learning in order to predict user activity, analysing emotion and speech to personalize the outcome of the system based on user behaviour and preferences. The older adult receives personalized notifications on how to best manage her/his health along with recommendations, advice, and tips (e.g., “today is market day”), including psychological and emotional support. The recommendations are not interactive and are received as short messages with recommendation text. Recommendations can be based on the tracking of this shared care path, and positive messages can be sent (e.g., instead of saying “you are not on target”, a notification might say “if you walk for another 12 min today, you will be on target” or “have you talked to a friend/relative on the phone today?”). Nudging recommendations are provided in an engaging and ethical way (e.g., showing a bottle of water with hour marks to promote water intake) in order to support self-management of comorbidities. The available recommendations are delivered through the virtual caregiver module. Currently, the available recommendations do not require affirmation by the user that the recommended action has been executed.

**Lifestyle monitoring:** this module collects user information about vital signs and biometric data, physical activity, food and nutrition, psychological state of mind, therapy adherence, and socialization. Biometric data are collected through connected devices such as digital blood pressure monitors, digital glucose monitors, digital pulse oximeters, sleep and stress monitors, digital dynamometers, etc. The Elder Care platform can integrate multiple commercial-grade medical devices for this purpose. In addition, The Elder Care platform supports manual data entry. Manual data entry can be executed by the older adult or by informal or formal caregivers and social care providers. In addition, the lifestyle monitoring module can collect user biometric data retrieved through smartwatch sensors (heart rate, sleep quality, and physical activity). However, description of the third-party devices and their level of interaction is beyond the scope of this paper. These data are not intend to be as accurate a measurement as provided by actigraphy devices. Instead, they provide an effective approximation of the user’s lifestyle which, when combined with PROMs, can offer a valuable monitoring tool.

**Smart Calendar**: this module enables older adults, the caregivers, and care professionals to set up different events in the foreseeable future (e.g., planned goals and activities before the next meeting, which itself is planned and entered into the schedule). Each event entry has a minimum level of required information: name of event, due date and time, occurrence (one time vs. recurring) and frequency (e.g., every three months), notification target (e.g., for the older adult, for care professionals, or for both), event reminders (e.g., sending an alert one or three days before event, 30 min before event, etc.), and notes related to the event. The older adult can enter their own events. Events can be updated, new times set, notes added, etc. Certain changes such as changing the schedule of the next visit can be valid only if both the elderly person and the care professional first approve them. In addition to events, the smart calendar permits flexible visualization of medication needs, the shared care plan, social activities, physical and diet plans, etc., in a time view. The smart calendar is connected with the recommender and alert modules to provide event reminders and to prompt scheduled activities. The older adult can define personal reminders such as family birthdays and other socialization-related events as well.

**Alerts**: this module provides preventive and emergency notifications for both care professionals and users. Management of alerts is determined based on the care team and the shared care plan, and the exact content of the alert and the triggering events can be modified by care professionals at will.

For care professionals, the following alerts are pre-set:

- As soon as a risk for frailty or pre-frailty is assessed for a specific adult, an appropriate alert is provided to the care professionals.
- In case of critical events (e.g., dangerous blood sugar levels), an alert is provided based on biometric data thresholds.
- In the case of an emergency (e.g., a fall), the system prompts an urgent alert to the caregiver (if applicable) and informs a nearby emergency centre.
- The self-assessment module uses combined lifestyle data from the lifestyle monitoring module to inform physicians in case of alerts, whereas the lifestyle monitoring module can send alerts as well based on expert-defined rules and collected data.

For elderly adult users:

- Alerts can be issued as a reminder to follow the care plan.
- Alerts can be sent based on calendar events (reminding of a visit, an event, family birthdays and other socialization related events, etc.)
- In case of critical events (e.g., dangerous blood sugar levels), based on biometric data thresholds the appropriate alert will be sent to the older adult based on their individual profile.

Finally, the alert system can send urgent alerts to the caregiver (if applicable) or even to a nearby Emergency Centre.

**Medication:** this module provides a medication and dosage list for older adult users, and checks for potential drug interactions. It is connected with the smart calendar in order to provide scheduled of medication intake and with the recommender in order to prompt medication adherence. Data about medication adherence are collected manually through the lifestyle monitoring module and depicted in a dashboard. In this module, medication adherence is tracked and the care team notified of the adherence status based on the information declared by the older adult user.

**Intelligent Dashboard and Analytics:** this module gathers data from other modules, external EHR, systems and biometric devices and sensors and provides an overview to care professionals. Several displays facilitate viewing of aggregated data and allow monitoring of progress on the most important clinical and social parameters in real time. The dashboard makes available aggregated data such as agile reporting of group of users by imputed variables, statistical analysis and graphical reporting of users, shared care plans, critical alerts, etc. The dashboard offers a single access to social and healthcare services and ensures real-time connection between users and caregivers to allow reporting of any problems that must be managed and routed to the appropriate professional. Machine learning and AI algorithms, including risk assessment and frailty algorithms, are used to analyse the data gathered by the Elder Care platform. Data are visually presented through the intelligent dashboard based on risks and other relevant classifications on an individual basis. The intelligent dashboard is modularized, and its various components are draggable on the screen. They can be adapted over time based on the types of information that are desired through a feedback loop from all involved stakeholders.

Finally, through the intelligent dashboard data can be selected, anonymized, and extracted for research purposes.

**Education and Information:** this module provides information material, educational resources, and training to support emotional, psychological, and physical wellbeing and empowerment. Training materials are provided in order to increase the skills of older adults as well as their caregivers and care professionals. The training content is selected from a list of available modules divided in three main topics: (1) soft skills for training emotion regulation strategies, effective communication and active listening, empathy, negotiation, and conflict resolution, resilience and self-motivation, loss and grief, etc.; (2) IT usage training on new technologies in order to leverage the potential of the system itself as well as to empower older adults and care professionals; and (3) training in acts of care such as self-care, health promotion, care techniques, etc. The training modules will include multi-media content (tutorials, videos, interviews, etc.) with special attention on demonstrations (e.g., how to use medical devices).

The presented content is dynamic, i.e., content is suggested depending on the user’s knowledge and capabilities as well as their accomplishments during training.

**Serious Games:** this module delivers serious games based on therapeutic goals anchored in the experiences of sharing, giving, and receiving and intended to increase the skills of older adults. Serious games are used to improve physical and mental fitness. The recommender is used to find a point where the intrinsic and extrinsic motivation of the user converge, i.e., where the user can enjoy a future desirable outcome (e.g., better mobility) made virtually present in the game. The principles of attention, active learning, feedback, and consolidation are already incorporated in the available serious games, and can be further personalized based on the user profile. Serious games can be prescribed by care professionals or recommended by the virtual agent in a suitable way considering the dose-intensity of training and the balance between practicing tasks using gaming systems and practicing functional tasks outside the simulated environment.

**Report generator:** this module supports different types of reports using information collected and processed from all available modules. The report module can be called from other modules to allow appropriate reports to be produced. For example, each time data are selected and analysed in the intelligent dashboard, these can be exported as a report. In addition, standardized reports are offered to help care professionals to adjust therapy and support decision making. Suitable reports for older adults are available as well, presented with a user-friendly design and understandable language and graphics. Multiple options for exporting the report are available (PDF, HTML), along with the option of printing the results directly.

### 3.3. Semantics and AI Tier

**Semantics and Data Lake**: semantic integration refers to the challenge of providing unified and transparent access to a collection of data stored in heterogeneous data sources using semantic models. During the last several years, ontologies have been used in order to integrate structured and semi-structured data. However, there is not a single correct way to model a domain, and several ontologies/standards/terminologies exist. Obviously, the amount of information available, the heterogeneity of the information, and the wide range of proposed ontologies dictate the identification of a solution able to handle all this information, especially in the context of the present Elder Care platform with its multiple devices, sensors, and self-assessment and data collection tools. As such, and based on experiences from projects like MyHealthAvatar [26], a modular ontology is used in the Elder Care platform as a global scheme to integrate all internal and external data, enabling a homogeneous view of all available and heterogeneous data. This enables uninterrupted access to all relevant information by the modules on the top as if all data were completely normalized, cleaned, and transformed to a single relational database [27].

**AI Tier**: this tier includes all intelligent algorithms used in the Elder Care platform. More specifically, it includes the risk models and the detection algorithms; risk stratification algorithms allow the identification of patients at risk of frailty or pre-frailty, and enable staging of users already in the frailty stage. There are currently a variety of risk models, such as the Fried Frailty Phenotype Framework [28], the Cumulative Deficit Model [29], the Frailty Risk Index, [29] and FIRE-MADE [30]. Based on the data available for each individual, a majority voting machine-learning algorithm is able to combine two or more of the aforementioned risk models in order to present an individual risk and staging analysis. In all cases, the final results are explained to the relevant health professionals by presenting both the output of the individual risk models used in each case and the parameters that most affected the prediction in each case. Care providers are then responsible for suggesting specific activities to the user such as changing the medication or care plan.

### 3.4. Interoperability Services

The Elder Care platform complies with all the requirements regarding interoperability, ICT standards, protection of personal health information, and interfacing with local systems. In fact, the solution can be integrated with any health system because it is built following the interoperability-by-design paradigm described in the new European Interoperability Framework (EIF) [16]. The Elder Care platform can apply the EIF interoperability principles at any setting. The Elder Care platform addresses legal interoperability by following existing legislation and data protection requirements in the countries where it is intended for use. Organizational interoperability is achieved by aligning the Elder Care platform with potential buyers’ group business processes, responsibilities, and expectations in order to achieve commonly agreed upon and mutually beneficial goals. The requirements of the user community are met by making the required services available, easily identifiable, accessible, and user-focused.

Semantic interoperability is achieved through the use of the available vocabularies and standards in use for data exchange, ensuring that data elements are understood in the same way by all communicating parties taking into consideration local needs. Data and information are treated as a public asset that should be appropriately generated, collected, managed, shared, protected, and preserved. The Elder Care platform implements all aspects of technical interoperability, including interface specifications, interconnection services, data integration services, data presentation and exchange, and secure communication protocols. The technical interoperability of the Elder Care platform is built upon the technical specifications of clinical sites in order to address the need to offer services in an integrated way, whereas full integration is possible with national and regional health care professional and patient portals for proper user and patient authentication and authorization as well as for the automatic retrieval of patient demographic data and other data based, e.g., on HL7 messages [31]. The Elder Care platform ensures technical interoperability by providing a data lake, gathering data from different types of data sources such as care professionals (e.g., provided medication), the mobile devices of informal care providers (e.g., answering a questionnaire with the elderly), non-clinical data sources (e.g., cultural agenda, weather, local news, information from any open data portal).

All of these data are made available and exploitable through ontologies/terminologies which enable homogeneous data access through standard application programming interfaces (APIs) to the rest of the platform components. In those APIs, standard open formats are used (JSON) in order to ensure that information can be consumed by both internal and external components as needed. Relevant information can be retrieved by external EHR systems as long as standard, open, and well-documented interfaces and APIs are used.

Given the sensitivity of the personal data stored in and used by such end-user applications, the definition and implementation of security components is fundamental to the platform design. The Elder Care platform must be compliant with existing European and National laws regarding personal data protection, security, and privacy. Following the secure-by-design principle during platform development, the Elder Care platform incorporates all EU regulations on privacy and data safety within all of its modules and features.

Finally, because cloud services are expected to be used for the hosting of the Elder Care platform, these need to conform to standards that support protection of personal data in the cloud as well as the quality of service and information security aspects of cloud computing and security management best practices, namely:

- ISO/IEC 27018:2019
- ISO 9001:2015
- ISO/IEC 27017:2015
- ISO/IEC 27001:2013

The Elder Care platform focuses on facilitating shared care plans for frailty management, promoting a self-aware and informed decision-making approach for older adults and their caregivers while compensating for the difficulties in the shared decision-making approach faced by clinicians. Older people can provide information about their conditions remotely without the need to travel to a specialist physician’s office for a consultation.

The platform follows the interoperability-by-design principle as based on the implementation of international standards such as HL7 and FHIR. It can interact with third-party systems such as devices and digital platforms based on the protocols that these systems support [32,33]. The platform can propose suggestions for a healthy lifestyle to the older adult user. These suggestions are based on the plan that the user has discussed and agreed upon with their healthcare and social care providers. The platform is a tool that allows different stakeholders to engage and interact while having common access to the same information. Based on responses to questionnaires and vital sign monitoring, care professionals can have a better idea about how the older adult is doing in their daily life. It is, however, the responsibility of care professionals and the older adult to work out shared care plans and engage in goal-setting. The platform does not automatically do this; rather, it provides information to facilitate decision support by the care professionals.

## 4. Discussion

In the past several years, information and communication technologies have been increasingly proposed as potential interventions to support older adults affected by several clinical conditions. Available results suggest that in general ICTs may impact the wellness of older people (e.g., improve their access to healthcare services, enhance their quality of life, empower individuals to better adhere to a healthy lifestyle, and support their social participation) [34,35]. Despite broad interest in the application of ICTs in the clinical management of the older adult, ICT application in the context of frailty in older adults has been poorly investigated in recent years [36].

Most of the ICTs developed for frailty usually focus on accelerometers and inertial sensors, dynamometers, and/or motion sensors, and generally aim at improving the evaluation of frailty using ICT to sharpen gait analysis [37], standardize the measurement of physical activity [38], support frailty detection and classification [39], and test the effects of rehabilitation [40]. Other studies evaluate the effect of telemonitoring to prevent frailty in older adults with clinical problems; however, these attempts are fragmented and have poor results in older adults [41] with respect to decreasing the rate of functional decline as measured by frailty states and mortality.

As such, available ICT-enabled services for frailty management mainly offer non-personalised information, are fragmented, and do not properly cover the population needs in a comprehensive way. Even services that allow for maintaining a health record (i.e., MS HealthVault, https://www.healthvault.com/ accessed on 27 December 2021) and exchanging experiences with other patients (i.e., PatientsLikeMe, https://www.patientslikeme.com/ accessed on 27 December 2021) on any disease, offer limited and non-personalized support. On the other hand, various apps for self-management are available, generally with a focus on a particular aspect of disease management such as medication management, drug–drug interaction, and serious games for anxiety therapy or physical activity. However, the available services are not designed to support shared decision-making and communication between patients and health professionals, and only a few are integrated into the electronic medical record systems of public health authorities.

Lack of data-sharing possibilities limits the capacity of clinicians to establish integrated care pathways, follow up on patient behavior, and support self-management initiatives. Technologies mainly exist as isolated solutions for generalized management of disease processes. Instead of simply adding ICT technologies to standard and traditional care practices, it is urgent to move towards effective strategies that could influence the culture of assisting frail subjects, educate and train specialists in the use of new technology, and foster transformation in the way care providers interact with patients and caregivers [42].

### 4.1. How Global Needs Are Addressed

The Elder Care platform described in this paper aims at the effective use of emerging technology for the delivery of person-centred integrated care and health services responsive to older people. The fact that the elderly are often frail and/or dependent can be dealt with by providing both they and their formal and informal caregivers the necessary tools to enhance their abilities and provide them with access to quality long-term care. The tools described here, when used either individually or in combination, have the capacity to both empower and motivate older adults to improve and maintain their independence, functional capacity, and health status as well as to preserve their physical, cognitive, mental, and social well-being.

Certain prerequisites involve the appropriate preparation of the workforce towards automation, the strengthening of community practice by sharing best practices, and the systematic provision of support to caregivers through appropriate policies that can accelerate the needed digital transformation of the relevant health, social, and care sectors.

The proposed platform addresses global needs by providing the following:

- Support to integrated health care delivery through the promotion of older adults’ active participation into her or his care plan, facilitating the adoption of integrated pathways of care that are more efficient, less costly, and that enable autonomy.
- Uninterrupted and secure sharing of information through, e.g., alerts, newsfeeds, etc., among all involved stakeholders, enabling communication of meaningful information across service boundaries, reducing unnecessary visits, and accelerating emergency handling responses, thus improving sense of security.
- Psychological and emotional support to mitigate emotional distress, loneliness and isolation while avoiding social exclusion, supporting independent living, and promoting healthy lifestyles and safe habits.
- Through appropriate education and training, engagement and technology acceptance are enabled, as older adults may face difficulties in using some technologies due to a low level of proficiency with digital skills.
- When appropriately combined, homogenized, and used, data analytics and AI technologies (through advanced machine learning and big data techniques) can be used to formulate a solid global health knowledge base.

### 4.2. Potential Value and Benefits to Stakeholders

The Elder Care integrated care platform addresses an important gap in the ICT industry, offering a complete digital solution supporting continuum of care for frailty prevention and management in older adults. It is a disruptive solution not currently available in the described form elsewhere in the market supporting integrated care pathways, shared care plans, and a collaborative environment among different stakeholders and is specifically designed for older adults and their needs, with an emphasis on knowledge sharing. The main value propositions and potential benefits of the Elder Care platform for each stakeholder group can be summarized as follows.

#### 4.2.1. Potential Benefits for Elderly People

- Empowerment of older adults to better manage their condition and their life by being offered a digital solution for the prevention and management of frailty that encourages independent living and wellbeing and supports self-management as well as personalized training about frailty in close cooperation with healthcare and social care professionals and caregivers.
- Increased sense of safeness at home despite functional limitations due to health conditions, and improved perception of loneliness and isolation due to better managing critical events/emergencies such as falls or acute episodes based on the use of preventive and emergency alerts.
- Preventing loneliness and isolation by using a digital tool that supports psychological and emotional support in close cooperation with informal caregivers, formal caregivers, social workers, and other professionals.
- Prevention and comprehensive management of functional and cognitive decline and psychosocial frailty and isolation and/or the perception of loneliness and isolation due to multimodal interventions supported by the solution.
- Increased role of older adults in their own care and effective self-management.
- Easy access to specialized healthcare and group support with people with the same or similar conditions.
- Improved health outcomes and quality of life due to better frailty management provided in cooperation with their attending healthcare and social care professionals based on a personalized shared care plan, optimized drug therapy, personalized monitoring, coaching for a healthy lifestyle, training, and improved adherence to therapy.
- Reduced number of visits to healthcare providers’ facilities as compared to current care.
- Overall improved patient experience regarding the level of frailty management and integration of care among care givers.

#### 4.2.2. Potential Benefits for Healthcare Professionals and Social Workers

- Empowerment of healthcare professionals and/or social workers to better manage pre-frailty and frailty by using a digital tool that supports systematic routine screening for pre-frailty stages in at risk older adults in clinical practice based on frailty algorithms that embed available existing data and data from other sources, predictive methods based on artificial intelligence, and by having access to an SCP and easy-to-use summaries (dashboard), allowing prioritisation of cases and faster reaction to patient needs.
- Better information exchange and improved communication and decision-making through the use of an SCP and dashboard as well as artificial intelligence based on users’ clinical and laboratory results and data derived from self-assessment (wearables and self-report), accomplished activities, etc.
- A better-informed, prepared, and trained workforce through use of a digital solution that supports integrated pathways of care.
- Reduced time required for healthcare professionals and social care workers to manage a person with frailty due to frailty algorithms, AI-based predictive methods, SCP, dashboard, etc.
- An improved experience for healthcare and social care professionals in managing patients with frailty.

#### 4.2.3. Potential Benefits for Healthcare and Social Care Systems

- Seamless, integrated frailty care approach covering the whole journey of citizens/patients through the health continuum of detection, diagnosis, management and home care based on integration of the solution with various sources of data, including patient-generated data processed through medical devices and the procurer’s existing electronic health records, personal health records, etc.
- Improved healthcare processes regarding frailty management based on a seamless and integrated frailty care approach.
- A shift to patient-centric intervention strategies that support the promotion of independent living of the ageing population by matching older adults’ needs, preferences, and limitations with existing care pathways and SCPs.
- Lower costs of healthcare and social care services related to frailty (e.g., reduced hospital admissions/emergency department visits, bed occupancy, laboratory exams, etc.) due to better management of pre-frail and frail patients at the community level, including detection of pre-frail and frail patients, provision of psychological and emotional support, functional decline support, and managing of critical events/emergencies.
- Managing demand and increased sustainability/cost-effectiveness of health and social care by optimizing resources, systems and societal costs associated with ageing through the promotion of cooperation between all involved stakeholders (healthcare professionals, formal caregivers, informal caregivers, and social workers).
- Improved public health for the ageing population thanks to prevention and better management of frailty and related conditions.

### 4.3. Main Challenges for Platform Implementation

Major challenges for implementing the integrated platform for the support of coordinated elderly care are related to the solution design phase, the prototyping phase, and the pilot implementation phase. The inclusion of elderly people, informal care givers, and clinicians in the design and development of the solution is very important as this can greatly impact their involvement in the use of the solution. Another critical factor in the design phase is understanding the patient journey and the related healthcare and social care processes and their integration of the solution into existing workflows. A user-centric design that utilizes an agile working model to develop software in short iterative cycles followed by testing and adaptation is a prerequisite for the development of an effective digital health solution.

Other challenges that need to be addressed in order to build a successful and operational digital health solution include:

- Conservatism and reluctance to adopt new technologies on the part of healthcare providers.
- Integration with IT systems of healthcare and social care organizations can encounter many potential problems.
- Definition of a clear value proposition that addresses unmet stakeholder needs.
- Combining healthcare and technical expertise to develop a robust and compliant solution that protects privacy, complies with regulations, and achieves interoperability along with reliable production.
- Demonstration of the safety and efficacy of the solution through clinical trials and/or real-world use, as well as gaining and maintaining regulatory approval, are critical factors necessary to support both the credibility of the solution and market access.
- This previous challenge is highly related to the appropriate certification that such a platform should receive (CE mark) as well as to medical device regulation (MDR) compliance. As the platform includes AI algorithms, it should additionally be compliant with EU Artificial Intelligence Act, ensuring that all required regulatory conditions are appropriately met.
- Validation of the potential impact of the solution (i.e., the value propositions) and the profitability of the solution.
- Achieving fit between the value proposition of the integrated platform and the needs and expectations of users.
- Developing a sustainable and scalable business model that supports the operation of the solution in real-world environments.

## 5. Conclusions

This work presented the essential elements needed for the provision of an integrated platform to support the coordinated care of the elderly related to frailty management. It has been based on the parallel evolution of separate concepts and products into a single platform that can be used either as-is or in conjunction with complementary tools and services for the provision of coordinated elder care. Digital solutions can support integrated care for older adults and address the unique challenges of frailty. Such solutions need to be reliable, efficient, holistic, and responsive in order to facilitate the early detection of frailty, foster prevention of disability and adverse events, and minimize hospital admissions. Digital solutions can contribute to a better understanding of frailty and its progression by collecting information about psychosocial factors and gender dimensions. A solid knowledge base introduces the potential of moving understanding of frailty and related health and care provision forward.

Digital platforms need to incorporate a holistic approach in order to address frailty prevention in terms of both physical condition and psychosocial risk factors. Innovative solutions can play a role in mitigating feelings of loneliness and isolation while supporting mental health and independent living. They can play an important role in promoting healthy lifestyles and safe habits. User-centred digital healthcare interventions tailored to individual needs can prove more efficient in slowing the impact of frailty than currently-available intervention programs while enabling user empowerment by offering tools for self-management through education and training, enhancing feelings of security and support. Digital platforms are fundamentally necessary for collecting data which, based on appropriate consents and anonymization, can then be further used for research purposes. Methodological and standardized research on different aspects of frailty will support connecting findings into an evidence-based roadmap for different data-driven use-case scenarios. Furthermore, digital solutions must focus on being cost-effective, sustainable, and affordable for purchase costs as well as for maintenance and operational costs.

As a future step, the authors intend to explore how to optimally categorize the information collected and the appropriate intervals of collecting such information in order to render the platform as unobtrusive as possible. To this end, the classification of information as necessary, nice to have, and advanced or detailed will enable novice users to provide only basic information, and as they become more proficient with the system to gradually provide more detailed information as needed.

The incorporation of qualified training materials and quality assurance need to be preserved, and the existence of appropriate regulations that can provide the necessary incentives for healthcare providers to use the ICT platforms is necessary as well. To ensure use in clinical practice, digital platforms need to be certified (CE mark), MDR compliant, interoperable, and in line with clinical evidence. Innovative solutions can only support integrated care if they can be customized to interact with existing solutions in different health and care systems. The effective continuity of care across a range of health and care services can be facilitated through the communication of meaningful information across service boundaries.
